# Supplementary material for: Rescue of Iqsec2 Knockout Mice with Human IQSEC2 Adeno-Associated Virus Mediated Gene Therapy
Source: Int J Mol Sci. 2025 Aug 27;26(17):8311. doi: 10.3390/ijms26178311 (PMC12427686; doi:10.3390/ijms26178311)
Supplement: Supplementary file 1 [file ijms-26-08311-s001.zip › ijms-3781111-supplementary.pdf]

Supplementary Figures

Figure S1. Growth curves in Het KO female mice with rat IQSEC2 and human IQSEC2 AAV.

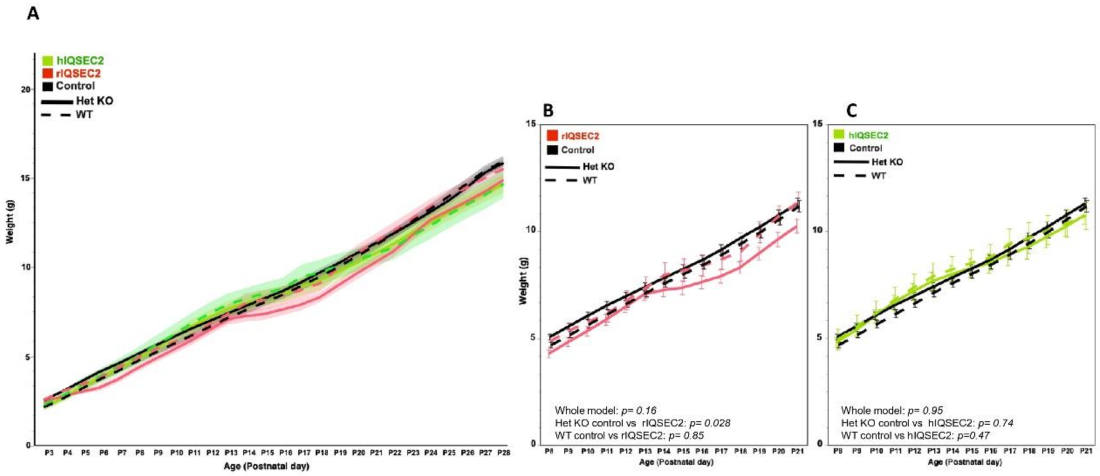

Figure S1. No benefit of IQSEC2 AAV on the growth of neonatal female heterozygous *Iqsec2* knockout mice.

A. Growth curves of female mice. Female *Iqsec2* heterozygous knockout (KO) mice are indicated by solid lines and female *Iqsec2* wild type (WT) littermates are indicated by dotted lines with the three treatment groups indicated by: Control- Black; rIQSEC2- Red; hIQSEC2 – Green. The shaded region for each curve is +/-SE of the mean.

B. Growth curves of female heterozygous KO and female WT mice receiving AAV rIQSEC2 (red) or control (black) treatments. MANOVA with repeated measures p = 0.16 (whole model); pairwise comparisons between PND 8-PND 21: KO (rIQSEC2 vs. Control) p=0.028; WT (rIQSEC2 vs. Control) p=0.85. Control-WT (n = 25), Control-KO (n = 54); rIQSEC2-WT (n = 11); rIQSEC2-KO (n = 17).

C. Growth curves of female heterozygous KO and female WT mice receiving AAV hIQSEC2 (green) or control (black) treatments. MANOVA with repeated measures p=0.95 (whole model); pairwise comparisons PND 8-PND 21: KO (hIQSEC2 vs. Control) p=0.74; WT (hIQSEC2 vs. Control) p=0.47. Control-WT (n = 25), Control-KO (n = 54); hIQSEC2-WT (n = 6); hIQSEC2-KO (n = 11).
